# Testing 3 Modalities (Voice Assistant, Chatbot, and Mobile App) to Assist Older African American and Black Adults in Seeking Information on Alzheimer Disease and Related Dementias: Wizard of Oz Usability Study

**DOI:** 10.2196/60650

**Published:** 2024-12-09

**Authors:** Cristina Bosco, Fereshtehossadat Shojaei, Alec Andrew Theisz, John Osorio Torres, Bianca Cureton, Anna K Himes, Nenette M Jessup, Priscilla A Barnes, Yvonne Lu, Hugh C Hendrie, Carl V Hill, Patrick C Shih

**Affiliations:** 1 Luddy School of Informatics, Computing, and Engineering Indiana University Bloomington, IN United States; 2 School of Nursing Indiana University Indianapolis, IN United States; 3 School of Public Health Indiana University Bloomington, IN United States; 4 School of Medicine Indiana University Indianpolis, IN United States; 5 Alzheimer's Association Chicago, IL United States

**Keywords:** older African American and Black adults, Alzheimer disease and related dementias, health literacy, Wizard of Oz, voice assistant, chatbot, mobile app, dementia, geriatric, aging, Alzheimer disease, artificial intelligence, AI, mHealth, digital tools

## Abstract

**Background:**

Older African American and Black adults are twice as likely to develop Alzheimer disease and related dementias (ADRD) and have the lowest level of ADRD health literacy compared to any other ethnic group in the United States. Low health literacy concerning ADRD negatively impacts African American and Black people in accessing adequate health care.

**Objective:**

This study explored how 3 technological modalities—voice assistants, chatbots, and mobile apps—can assist older African American and Black adults in accessing ADRD information to improve ADRD health literacy. By testing each modality independently, the focus could be kept on understanding the unique needs and challenges of this population concerning the use of each modality when accessing ADRD-related information.

**Methods:**

Using the Wizard of Oz usability testing method, we assessed the 3 modalities with a sample of 15 older African American and Black adults aged >55 years. The 15 participants were asked to interact with the 3 modalities to search for information on local events happening in their geographical area and search for ADRD-related health information.

**Results:**

Our findings revealed that, across the 3 modalities, the content should avoid convoluted and complex language and give the possibility to save, store, and share it to be fully accessible by this population. In addition, content should come from credible sources, including information tailored to the participants’ cultural values, as it has to be culturally relevant for African American and Black communities. Finally, the interaction with the tool must be time efficient, and it should be adapted to the user’s needs to foster a sense of control and representation.

**Conclusions:**

We conclude that, when designing ADRD-related interventions for African American and Black older adults, it proves to be crucial to tailor the content provided by the technology to the community’s values and construct an interaction with the technology that is built on African American and Black communities’ needs and demands.

## Introduction

### Background

As the US population is expected to grow older [1], the ethnic and racial composition of the United States will become more diverse than ever before [2]. Meanwhile, neurodegenerative disorders such as Alzheimer disease and related dementias (ADRD) remain one of the most common health conditions among older adults [3,4]. In line with current projections, the number of people worldwide that have dementia will rise to 82 million by 2030 and to 52 million by 2050 [3,5]; hence, it will become one of the most common health conditions among the geriatric population [3]. In addition, ADRD not only deeply impacts those diagnosed with it, but it has been found to also take an extremely emotional, physical, and cognitive toll on the people around the patient with ADRD [3,5], becoming a serious health and social concern. Moreover, studies have found that African American adults tend to be twice as likely to develop ADRD compared to any other ethnic group [6-8] as African American individuals present lower levels of ADRD health literacy [9] than other populations.

This discrepancy is due to historically unjust power structures [10] and discriminatory practices and policies [11] that have systematically limited this population’s access to the skills and the resources required to seek, obtain, process, and apply health information.

The term *health literacy* does not only refer to information about symptoms and preventive measures; rather, it encompasses all the information concerning the onset, development, and management of a specific disease. Therefore, health literacy includes information about help, support networks, and financial and transportation resources available to patients and their families [12]. Health literacy is extremely important in the treatment and management of a disease as having low health literacy is highly correlated with poorer health treatments and outcomes [13-15].

While a large body of work has focused on developing and testing ADRD health technology to improve health literacy among White older adults [8], those same technological interventions tend to be less successful, useful, and adopted among African American and Black communities. From prior work on the reasons for this discrepancy, it has emerged that health technological interventions can be successful among this population only when they are culturally relevant and infused with cultural values [16-20] and when they are co-designed and tested with the communities themselves [4,21-23]. Thus, it proves to be crucial to develop and test health technology that is specifically designed and tailored for the African American and Black populations centered on their unique needs and overcoming their unique challenges. The same assumption can be applied to ADRD-specific technology, which, to be successful, adopted, and perceived as useful by African American communities, must be designed specifically for these communities and be tested solely with African American participants.

This study built on this assumption and previous findings by focusing its efforts on testing an ADRD health technology developed using 3 different modalities—mobile app, chatbot, and voice assistant—with 15 older African American and Black adults. This allowed us to gather culturally specific design insights concerning the unique needs and challenges of African American and Black communities when using ADRD technology. In addition, this study addressed health disparities by assisting African American and Black communities through bridging the health literacy gap.

### Related Work

Addressing the low health literacy levels among African American and Black communities necessitates an understanding of their information-seeking behaviors and the obstacles they face in accessing health-related information. Some studies have identified that African American adults present lower levels of ADRD health literacy than White older adults [12,24], with some older African American adults holding inaccurate beliefs concerning ADRD (eg, believing that ADRD develops with normal aging) [25].

Despite the widespread availability of web-based health information, African American individuals exhibit the lowest use of digital tools for accessing such information compared to other ethnic groups in the United States [26]. This discrepancy is particularly pronounced among older African American adults with low incomes, who face additional barriers due to limited digital access and skills [27,28].

Few studies have investigated how older African American and Black adults use digital tools when seeking health information [29-31]. They primarily search for information related to specific diseases and their treatments on the web, but they often lack the tools and resources to assess the credibility of the information they find [31].

Older African American adults express a preference for information coming from trusted sources within their community, such as friends, doctors, family members, and community organizations [29,32]. The importance of the community in promoting health equity and empowering older African American adults to make informed decisions has been shown by recent research, wherein the most effective health interventions were the ones centered on the community with targeted education and support programs [9,10,33].

Given the low literacy observed among older African American adults and the health disparities impacting this population, recent studies have focused on how technology can become a tool to improve health literacy and access to health care and achieve better health treatments. Most of these studies have emphasized the importance of integrating this population into the design and testing process as doing so can ensure cultural relevance as well as improving the perceived usefulness and efficacy of the tool [23,33]. One way to integrate the population has been to include the community in the process. This can assure the collaboration and participation of African American individuals of a low socioeconomic status in envisioning health-related technologies [19,21,22,34].

In addition, from recent studies, the importance of integrating the sociocultural context and values of the community into the design of the technology has emerged. Indeed, cultural factors have been found to play a crucial role in one’s health, impacting attitudes toward health and health-related behaviors [16-20]. In addition, the cultural context with its values and beliefs impacts the way in which health technology is perceived, evaluated, and adopted [19]. For example, due to perceived racist treatment [35], historical mistreatment of their community [36], and discriminatory experiences in hospitals [37], African American adults have lower levels of trust toward the US medical system and medical research [38], leading to more skepticism toward health interventions and the technologies involved [19].

Mobile apps infused with African American cultural values have been found to be successful in increasing mindfulness behaviors and self-efficacy concerning health goals [33,39]. Similarly, culturally tailored mobile apps have shown clinically relevant outcomes in improving self-care maintenance among African American individuals with specific health conditions [23,40].

While all the aforementioned studies [23,33,39,40] focused on one way of interacting with the technology, more recent studies have shown the opportunities of designing new forms of interaction and technologies for aiding African American individuals in their health information–seeking process [34,41,42].

One of these innovative ways is the use of conversational agents as the core interaction modality of a health technology. Compared to mobile apps, conversational agents such as chatbots and voice assistants present some key advantages: they can provide personalized health information and support adapted to the user’s needs [5,34]; they can resemble a more direct and natural interaction, fostering adoption and use among a more technology-skeptic section of the population, which often tend to be African American individuals [19]; and, finally, they can be used by people with limited mobility, vision, and reading skills [43-45].

However, their adoption among older adults faces complexities. While some are common among older adults of other ethnic groups, such as issues of reliability, privacy, and autonomy loss [46-48], older African American adults experience unique challenges when engaging with voice assistants. Indeed, older African American adults experience frustration with voice assistants such as Google Home because of language comprehension issues that force them to code switch to access the tool [49]. In addition, often, African American and Black individuals lack the digital expertise or the infrastructure to access and use these conversational agent systems [23].

These studies [23,34,41,42,49] show how, while conversational agents such as chatbots and voice assistants offer promising avenues for assisting older African American adults in accessing health information [34,41,42], their adoption among this population faces challenges related to digital accessibility [23] and language comprehension [49].

These studies show the potential of conversational agents and mobile apps in assisting African American and Black individuals in accessing health information, as well as the unique challenges faced by this population when engaging with conversational agents.

From the aforementioned review of the literature, it appears evident that designing technology for older African American adults requires active collaboration with the target population and a deep understanding of their sociocultural context and cultural values. By adopting these 2 strategies, hence establishing a collaboration with the community and acquiring an in-depth understanding of the social-cultural context, it becomes possible to design culturally relevant technology infused with African American cultural values with the involvement of African American communities, which can successfully achieve better health outcomes for this population and help bridge the health access and treatment gap.

### Rationale of This Study and Objectives

While some recent papers have begun exploring technology interventions for the African American population, none of these studies have provided insights on how to design specifically ADRD-related technology for this population considering how the information content and the interaction with the tool should be tailored to meet the needs of this population. Given that ADRD is a serious and common health concern among the African American and Black communities [6-8], and given that ADRD health literacy is extremely low among this population, leading to poorer health outcomes and treatment [13-15], designing a technological intervention that provides ADRD-specific health information tailored to the needs of this population proves to be essential.

Our study contributes to this space by investigating how the interaction with an ADRD health information tool should be designed for older African American and Black adults by testing 3 different modalities—chatbot, mobile app, and voice assistant—as health interventions. We decided to test 3 different modalities to gather specific insights on how each modality is perceived and evaluated by older African American and Black adults. Indeed, these modalities have different affordances depending on the users’ own skills, capabilities, and preferences regarding the purpose of the interaction and the context of use [50].

Thus, by differentiating each modality and testing it separately, it becomes possible to gather data concerning the use of each modality in accessing ADRD health information; each modality’s affordances; each modality’s limitations and constraints; and, finally, each modality’s opportunities and spaces for improvement. Differentiating the 3 modalities allowed us to dig further into how each modality of interaction is used and how it is evaluated and perceived by this specific population. This is the first study to explicitly use a multimodality approach to investigating a health technological intervention aimed to assist older African American and Black adults in seeking ADRD information. In addition, differentiating the 3 modalities guaranteed the possibility of investigating how ADRD content intersects with each specific interaction modality and how the content should be presented to be consistent with the modality and vice versa.

Notably, our research goes beyond merely providing health information on ADRD symptoms and diagnosis to also include information concerning doctors’ availability, financial resources, and community events following the definition of health literacy offered by prior work [12].

Through the testing of these varied interaction modes of accessing ADRD-related information, this study aimed to uncover (1) the unique needs and challenges of older African American and Black adults seeking ADRD-related health information using 3 different technological modalities (chatbot, voice assistant, and mobile app); (2) the role that technology can have in improving health literacy; and (3) the way in which health technologies such as chatbots, voice assistants, and mobile apps are perceived and evaluated by older African American and Black adults by testing them using a Wizard of Oz methodology.

To obtain these findings, we conducted a usability session in a state within the United States. We gathered 15 participants and tested each technological modality. Then, we analyzed the data gathered (ie, the transcripts obtained by recording the participants’ inputs and insights). We concluded that, when designing ADRD-related interventions for African American and Black older adults, it proves to be imperative to focus on 2 aspects: the information provided and the interaction constructed. Regarding the information, it should be credible, community-centered ADRD information and accessible to all. Regarding the interaction, it should be designed to be time efficient, fostering users’ control and representation.

## Methods

### Overview

Participatory methods that prioritize user needs and experiences are crucial when building tools and interventions for vulnerable populations such as older African American and Black persons seeking information on ADRD. When it comes to including people with cognitive impairments and ensuring that solutions are both accessible and adapted to meet their individual needs, co-design and design thinking have shown to be especially successful [4]. Moreover, research has shown how digital technologies might improve the quality of life of individuals with dementia [5]. On the basis of these ideas, we used the Wizard of Oz technique to simulate system interactions and improve the user interface through real-time responses, allowing for more iterations before full implementation.

### Recruitment

Throughout this study, there was an emphasis on collaborating with the African American and Black community. This collaboration was achieved by organizing events and seminars in local churches, community centers, town halls, and libraries and creating a monthly newsletter with consistent updates on the research. In addition, a community advisory team board was created, and a monthly meeting between the latter and the researchers was organized. Finally, every design decision made by the researchers was discussed with and changed based on the inputs and opinions of the community advisory team board.

Participants were recruited through 2 community engagement specialists by organizing events and seminars on ADRD in community locations such as local places of worship of different faiths, local chapters of the Alzheimer’s Association, and local chapters of the Yong Men’s Christain Association. In addition, people who attended those events were asked to sign up for a monthly newsletter, which provided information about upcoming events and participating in this research. Moreover, recruitment was also conducted through word of mouth. Testing took place in the months of March 2023 and April 2023. There were 2 inclusion criteria: being aged >55 years and being part of the African American and Black community.

Before the workshops, participants were presented with an informed consent form and were asked to complete a questionnaire collecting demographic information (age, educational level, employment status, and history of ADRD in their family). Given that the goal of this study was to create a tool to improve ADRD health literacy among the African American and Black population in general and not people who are personally diagnosed with ADRD, all the participants reported good cognitive health and showed no signs of cognitive decline.

Completing the questionnaire was voluntary and did not influence participation in the design workshops; thus, in some cases, some parts of it were not completed. An overview of the demographics of the participants who attended the session can be found in Table 1.

**Table 1:** Demographic information of the 15 participants of this study, showing their gender, age, educational level, and employment status and whether they had a history of Alzheimer disease and related dementias (ADRD) in their family.

Participant number	Gender	Age (years)	Educational level	Employment status	History of ADRD in their family
1	Woman	71	Some college	Retired	Yes
2	Man	73	Some college	Retired	Yes
3	Woman	67	Associate’s degree	Retired	Yes
4	Man	68	Some college	Retired	Not reported
5	Man	56	Some college	Retired	Not reported
6	Woman	79	Bachelor’s degree	Retired	Not reported
7	Man	62	Some college	Employed full time	No
8	Man	80	Bachelor’s degree	Retired	No
9	Woman	62	Bachelor’s degree	Employed full time	Yes
10	Woman	64	High school	Employed part time	Yes
11	Woman	68	Some college	Retired	No
12	Woman	70	Bachelor’s degree	Retired	Yes
13	Woman	69	High school	Retired	No
14	Woman	68	Associate’s degree	Retired	No
15	Woman	58	Some college	Not reported	Yes

### Ethical Considerations

This study received the approval of the Indiana University institutional review board (approval number 12241). Participants were presented with an informed consent sheet before the study, and they were reminded of their right to withdraw at any time throughout and after the study. Participants were compensated with a US $50 gift card for taking part. Participants’ privacy and confidentiality were protected by anonymizing the data, changing the wording of the quotes presented in the Results section, and deleting all the audio recordings after the transcriptions were completed.

### Study Design: Wizard of Oz Structure

This study used a Wizard of Oz methodology, which allows researchers to present technologies to participants as semifunctional even though they are not fully developed. The Wizard of Oz methodology is highly used within the human computer interaction (HCI) communities, in particular when testing prototypical versions of voice assistants and chatbots [13,20]. This allowed the researchers to more thoroughly test the designs of these technologies and receive critical feedback from community members before potential further development.

Data collection took place in March 2023. The session was structured in the following way. Each modality (app, chatbot, and voice assistant) represented a station—station A was the mobile app, station B was the chatbot, and station C was the voice assistant. Participants were asked to rotate from one station to another every 30 minutes.

Thus, at the end of the 30 minutes, participant 1, who started at station A, had the chance to go to either station B or station C; participant 2, who started at station B, had the chance to go to either station C or station A; and participant 3, who started at station C, had the chance to go to either station A or station B. This rotational design was created to negate the influence of the order of interaction with the modalities on participants’ responses as much as possible.

At each station, a facilitator and a person responsible for the Wizard of Oz study were present from the research team. For each participant, the testing session of all 3 tools lasted approximately 1 hour and 30 minutes. Participants were asked to complete 2 tasks during each session. For the mobile app, the tasks were the following: (1) you want to read other people’s experiences to find some strategies to dress your loved one diagnosed with ADRD in the morning if they are resistant, (2) you want to learn about early signs of Alzheimer disease. For the chatbot, the task were the following: (1) you want to find a community support group near you to help you learn about ADRD and obtain emotional support, (2) you want to look for transportation help for your mother with ADRD. For the voice assistant, the tasks were the following: (1) you want to find some ADRD specialists near you who accept new patients, (2) you are interested in knowing whether brain health is important to prevent the development of Alzheimer disease. You want to check if it is true. For the voice assistants, the system would provide the same outputs for all the participants, which were as follows: for task (1), “Perfect! Let me ask you a few questions to help me out in this search for you. Are you looking for a doctor in your area?”; and for task (2), “According to the Alzheimer’s Association, keeping your brain active is important to prevent the development of Alzheimer. This is supported by the National Insitute of Health. Did I answer your question.”

Each modality presented slightly different tasks as each task was designed to be coherent with each technological modality; however, the tasks referred to 2 main areas across all modalities: seeking for community help and support and seeking for medical information concerning ADRD. All interactions were crafted to be consistent among participants.

Given that the goal of this study was to evaluate the usability of the 3 technological systems, we did not test the efficacy of our intervention concerning the level of ADRD literacy before and after the study.

The voice interaction as well as the chatbot always started by greeting the user as follows*—*“Hello! I am Lola, your virtual assistant for the CARE platform. How may I help you?”—followed by a consistent interaction according to the user’s inputs. Each participant received the same outputs across the chatbot and voice assistant modalities.

During the tasks, participants were asked to think out loud and share their opinions, thoughts, and impressions on the technology they were presented with.

### Data Analysis

The sessions were recorded and transcribed. All the data gathered were then analyzed thematically following the guidelines by Clarke et al [51]. Using an iterative, open, and axial coding process, 2 transcripts were used to identify emergent themes. From these codes, an initial draft codebook was developed and applied to the remaining transcripts using Taguette as a qualitative analysis software. When new appropriate themes emerged, they were added to the codebook until thematic saturation was reached and no new themes were identified.

Each transcript was analyzed and coded by at least 2 researchers to ensure an appropriate intercoder reliability. An initial data analysis was presented during an advisory team board meeting to share the preliminary findings with members of the community. Community members provided their feedback, and the researchers included their insights in the data analysis.

### Introducing Each Modality of Lola

#### Overview

In this section, we outline the process of designing prototypes for the usability testing sessions by focusing on 3 types of interactions: mobile app, chatbot, and voice assistant. We called this intervention Lola.

The design of these technology interventions relied on data gathered from 7 previously held focus groups and participatory design sessions conducted with older African American and Black adults. While discussing this previous research activity in detail is out of the scope of this paper, a summary will be provided for context. A total of 7 focus groups with 44 participants in total were held to discuss challenges and current strategies to achieving ADRD health literacy, whereas 7 subsequent participatory design sessions with the same groups engaged participants in brainstorming and collaborative design of potential solutions to improving ADRD health literacy. The results emphasized that older African American and Black adults are comfortable with using digital tools such as phones and computers to access information about ADRD. Participants discussed that they often needed information on caregiving best practices, ADRD prevention and diagnosis tactics, and community support.

#### Designing the ADRD Content for Lola

The implementation of the ADRD information across modalities was based on the conceptual framework built through the works by Guerriero Austrom et al [52,53], which offer a nonpharmacological approach and protocol for multifaceted care management of ADRD. This approach conceptualizes care as a collaborative process wherein both caregivers and patients are in need of support and help and wherein providing education and psychological support to both of them proves to be a crucial factor in the management of the condition. This results in providing general education about ADRD to allow people to obtain a general understanding of the medical conditions. It then involves psychoeducational and psychological support for caregivers, best practices to treat behavior disturbances in patients with ADRD, legal and financial advice, caregiver coping skills, and exercises with a guidebook.

The actual content present in the technological intervention was designed with the help of the community advisory team board, constituted by the research team; community engagement specialists and members of the African American and Black communities living in the research areas; and, finally, African American and Black ADRD professionals (eg, doctors, nurses, and ADRD researchers). Thus, the content was designed by the researchers with the help, suggestions, and insights of the community advisory team board as an iterative process wherein the research team presented the content to the board, received feedback and insights, made the implementations, and presented the revised content. In addition, some members of the community advisory team board were also members of the local chapters of the Alzheimer’s Association; hence, they could provide specific feedback thanks to their ADRD expertise. Finally, given the collaboration with the Alzheimer’s Association, the content of the intervention was modeled after the content presented in the association’s own database.

#### Lola as a Mobile App

**Figure 1:**
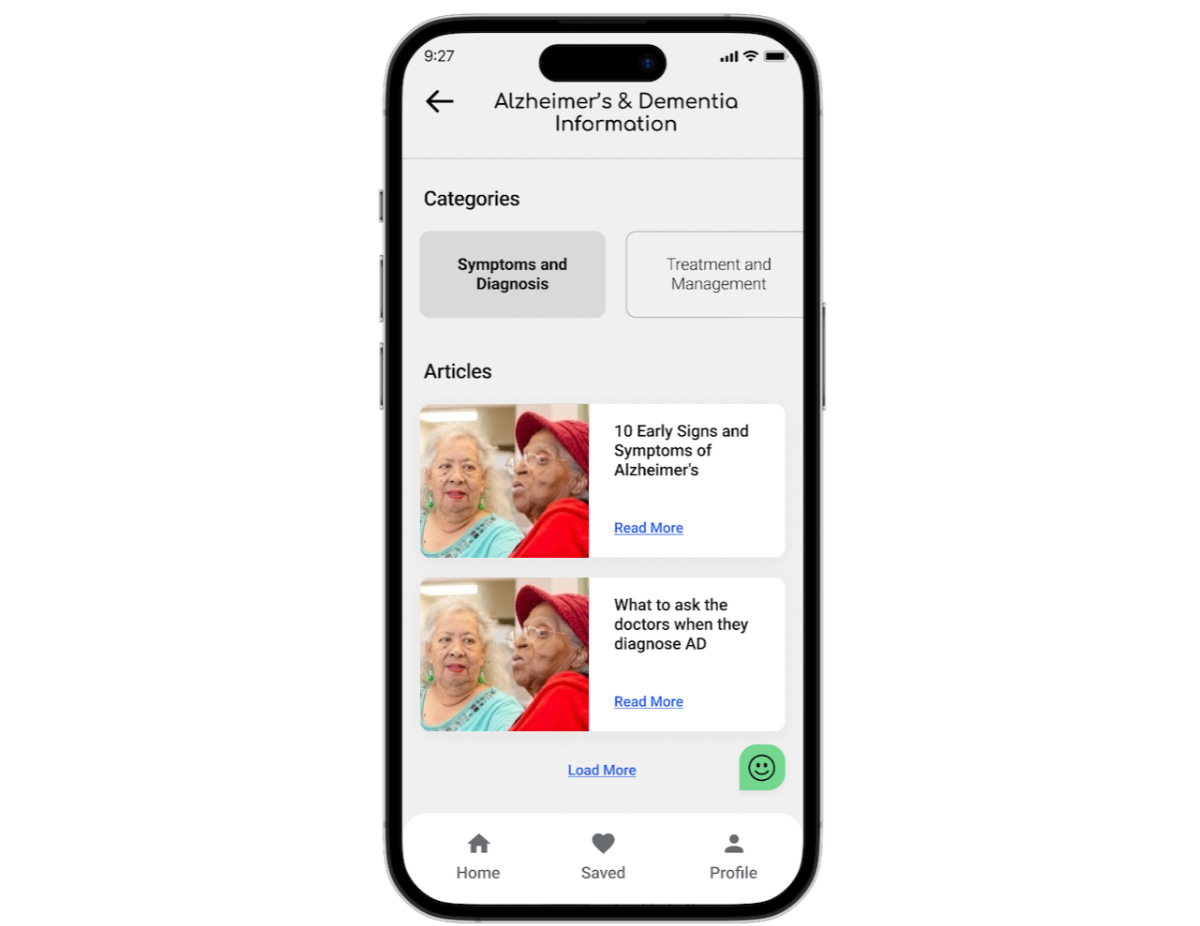
A screenshot showing the mobile app while navigating it (image with identifiable people adapted from Adobe Stock, and licensed by the authors).

The mobile app was prototyped using Figma (Figma, Inc) to allow researchers to observe participants’ interactions with a smartphone by performing 2 specified tasks. These tasks were to find a community forum and search for information related to ADRD. Participants’ starting point was the home page, which featured 2 main categories: “Alzheimer’s and Dementia Information” and “Community,” as shown in Figure 1.

To complete the first task, users navigated through the app to find the “Community Forum” under the “Community” category, seeking the experiences and tips of others coping with ADRD. In addition, to address concerns about trustworthy resources, nonprofessional users were distinguished from professional ones through badges such as “Medical Professional” or “Certified Caretaker.”

For the second task, participants were prompted to explore the “Alzheimer’s and Dementia Information” category, which included subcategories such as “Symptoms and Diagnosis,” “Treatment and Management,” “Risk factors and Prevention,” and “Caregiving Tips.” These subcategories provided up-to-date articles in different topics. From here, the user had to access a single article.

#### Lola as a Chatbot

**Figure 2:**
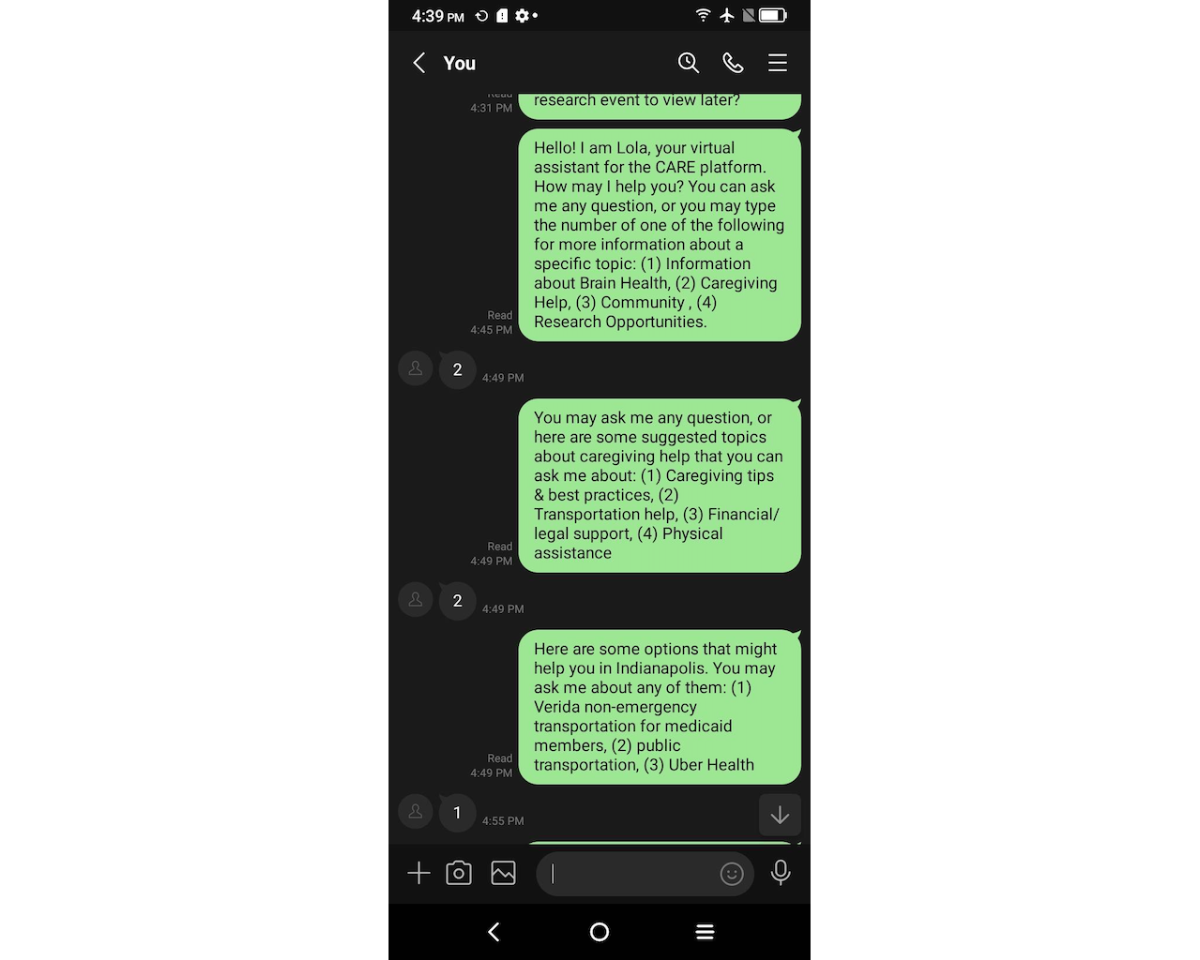
A screenshot showing the functioning of the chatbot.

Participants’ interactions with Lola as a chatbot began by receiving the introductory message on a smartphone: “Hello! I am Lola, your virtual assistant,” as shown in Figure 2. Further in the introduction, the chatbot explained its capabilities and offered participants 3 categories to choose from for more information: “Information about Brain Health,” “Caregiving Help,” and “Community.” Participants had the freedom to type in category names, category numbers, or custom responses to which the chatbot would respond. Within each category, subcategories directed participants to more information, such as “upcoming events” or “finding a support group” within the “Community” category, and when participants indicated that they no longer required assistance, the chatbot ended the conversation by responding, “I understand. Have a nice day!”

The chatbot version of Lola was prototyped using the Line app (LY Corporation). We established 2 separate Line accounts. One was operated on the smartphone device provided to the participant, whereas the other was managed by the researcher on a MacBook laptop using text prompts designed by the research team. These prompts were organized in a spreadsheet containing a predetermined conversation flow. The accounts were visually distinct, with the participant’s account having a default user profile picture and the researcher’s account featuring a Lola mascot. Standard responses were generated for each potential query, allowing researchers to select, copy, and paste the appropriate response into the chat based on the question posed by the participant. By having a researcher control the chatbot, the researcher was able to interpret participants’ messages and respond to them in a flexible manner using the predetermined responses even if participants interacted with the chatbot in ways that were outside the designed conversation flow. This method gave participants the impression of conversing with a real person.

#### Lola as a Voice Assistant

**Figure 3:**
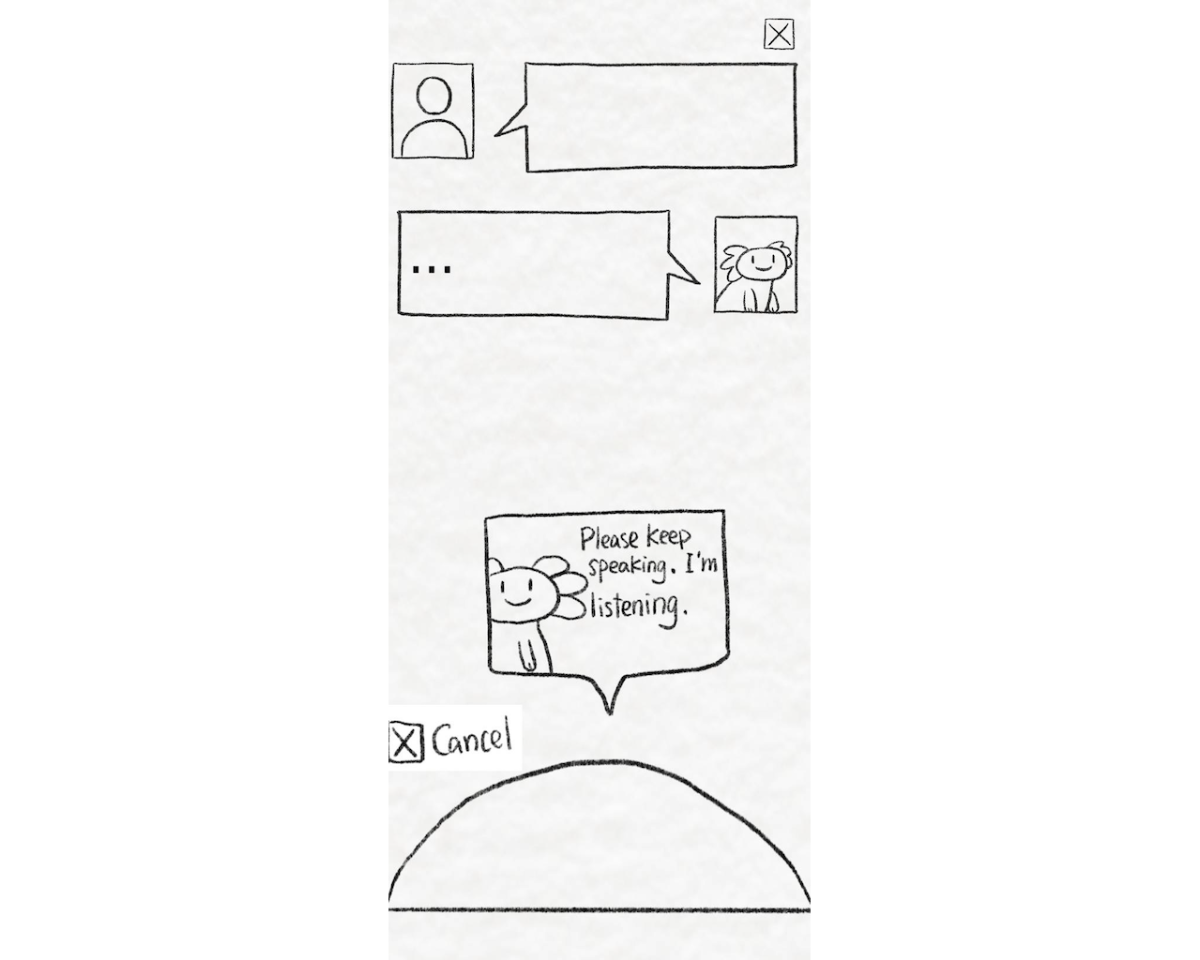
An illustration showing the functioning of the voice assistant.

The system for the voice assistant consisted of a smartphone and a Bluetooth speaker. The participant was asked to press the “start” button on the smartphone to initiate the interaction, but the voice of the voice assistant would only come out of the Bluetooth speaker. The smartphone was only provided to the user to foster a sense of comfort and active listening by showing messages such as “Please keep speaking. I’m listening” (which can be observed in Figure 3).

The flow for the voice assistant interaction mirrored that of the chat-based design. Lola initiated the conversation by introducing itself and its capabilities, guiding users on how to use the voice interface. The voice setup consisted of a Bluetooth speaker and a laptop controlled by a researcher for triggering responses according to the same predetermined flow used for the chatbot.

To ensure coherent researcher responses, we directed participants to ask a series of preset questions and used fixed answers to respond. All questions and answers were stored as text boxes in a custom program developed on a MacBook Pro, with the back end developed using the Unity engine (Unity Technologies). The question-and-answer flow was organized in a specific tagged sequence with an attached button at the end of each answer. Researchers were tasked with pressing the button to trigger the voice message stored within the program. The premade voice messages were made using a text-to-voice program, Murf AI, to generate audio messages of the prewritten responses.

## Results

### Overview

**Figure 4:**
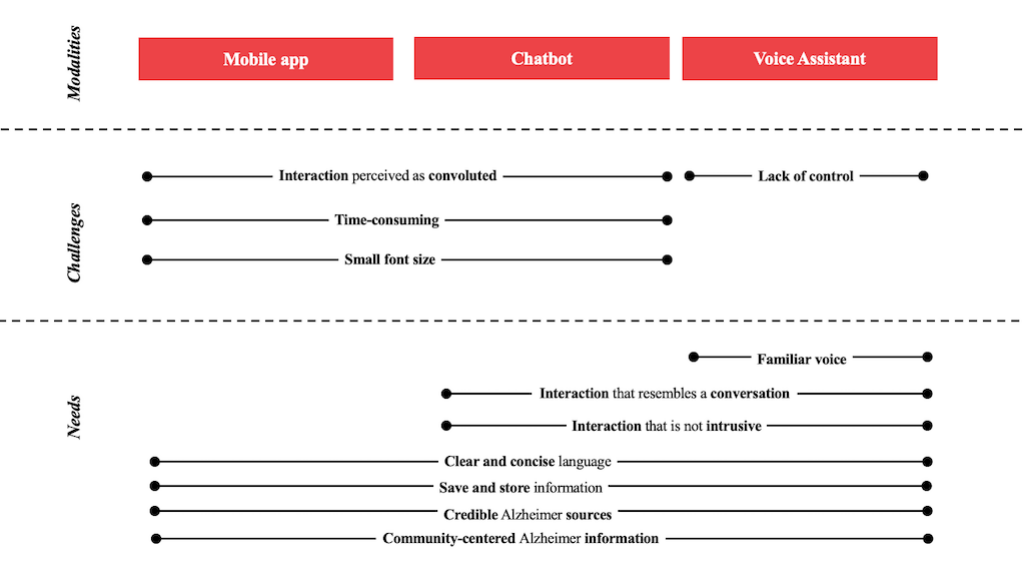
An overview of the results obtained in this study. ADRD: Alzheimer disease and related dementias.

From data analysis, the following 4 themes emerged: the importance of creating accessibility of information and accessibility of the technology; the importance of sources and contact information to elicit trust and credibility; the crucial role of the community; and the preference for a direct, time-efficient, and personified interaction. All themes were found in each modality. An overview of the major results can be found in Figure 4.

### Theme 1: The Importance of Creating Accessible Content and Technology

This first theme relates to the accessibility of the content and technology (Textbox 1). Concerning the accessibility of the content, a major request made by participants was the opportunity to store and save pieces of content obtained through each of the technological systems:

It would want to store it and save it, but I don’t know how it could be stored it, unless it is recorded somewhere.

P3; voice assistant

**The key findings of theme 1 on the importance of creating accessible content and technology.**

**To be accessible, content must have the following characteristics:**

- Ability to store and save pieces of information
- Ability to share with peers
- Larger font size
- Avoid using lengthy and convoluted language

**To be accessible, technology must have the following characteristics:**

- Be time-efficient in information seeking
- Not be excessive in tapping or steps
- Slow down the voice assistant
- Allow for asking the system to repeat

This need was not limited to the voice interaction, which could be perceived as more transient, but was also observed for the chatbot interaction and the mobile app:

Because, first off, I found this information, I may want to send, I may want to share it with my wife, or I may want to save it for later for further review. So, I would like to be able to save it and I would like to be able to share it. And I would definitely like to be able to print it.

P2; mobile app

In addition, participants complained that a small font size made it challenging for them to read the text, stating that small text could become a barrier for people with low-quality vision due to aging or medical conditions:

I think it’s small for older people. Yeah, it is small, and I’m nearsighted. So I can see up close. Some people could be farsighted, so they can’t see that close.

P3; chatbot

Another notable barrier that arose with the mobile app and chatbot was excessive length and complexity of the interaction flow. From the initial engagement to the point of providing the desired information, the process became cumbersome and time-consuming:

I don’t want to have to press 10 or 12 buttons to get where I want.

P4; mobile app

The usability testing revealed distinct issues with the chatbot and mobile app, but a different concern arose when participants interacted with the voice assistant. Many of them expressed dissatisfaction with the lack of control in the interaction, leading to feelings of being overwhelmed by the way in which the voice assistant provided information.

One participant specifically mentioned the desire for the system to repeat the piece of information it had just provided. He explained that this feature would be helpful, especially for individuals such as him who needed time to write down the given information:

Because there are some people that would rather write it out. So Lola [the voice assistant] needs to give me the time to do it. She needs to ask: Would you like for me to say it again? and then I can say, yes, a little bit slower.

P2; voice assistant

Another participant expressed the need to be able to ask the voice assistant to slow down to give her time to understand everything and write down all the relevant information:

I just want to make sure that I have the option of asking Lola [the voice assistant] to slow down. For some people, it is okay to be so quick, but for me, I just want to make sure I got all the information.

P3; voice assistant

Finally, across all modalities, participants expressed fear that the low digital literacy of their peers could impact their potential use of the tool:

Some of us used to work in the office, dealing with computers and the internet and all that. But some people may not have the same experience with digital technology.

P3; mobile app

This theme underscores the importance of designing and creating ADRD-related information that is accessible to all individuals, particularly emphasizing ease of use for older users. It highlights the significance of developing interactions across different modalities that cater to the unique needs and preferences of older African American and Black adults.

### Theme 2: The Importance of Providing Sources and Contact Information to Elicit Trust and Credibility

This theme emphasizes the significance of providing credible sources to instill trust in the technology and potentially enhance its use and success (Textbox 2). The inclusion of sources has a profound impact on how participants process, access, and evaluate health information:

Researcher: How do you decide if you can trust a piece of information?

P2 [mobile app]: Sources! if I am looking at what a doctor said, I want to know: their story, what makes them eligible, knowledgeable in this field.

**The key finding concerning theme 2, which deals with strategies to elicit trust and credibility.**

**The importance of providing sources and contact information to elicit trust and credibility**

Trust is elicited through the following:

- The inclusion of reliable Alzheimer disease and related dementias (ADRD) sources
- The possibility to get in touch with ADRD health professionals

A participant tied this need for sources with people in the African American and Black community being suspicious toward any help coming from outside their community:

I also find that in our community, we’re not open to new things. We think that there’s no help out there, but there’s a lot of help out there. But you have to be open to it. I’ve had different discussions with different people, and they don’t believe there could be help. So that’s why I say they have to have that information come from a trusted source, then They may read it, but it has come from a really trusted source.

P13; chatbot

Furthermore, participants emphasized their strong desire to have a specific source they could refer to enabling them to personally get in touch with the organizers of the ADRD-related events or the organization offering the service:

Lola [the voice assistant] gave me the phone number. So, if I had any other questions, I could actually talk to the person in charge of the research event.

P14; voice assistant

This theme highlighted the importance of not only trustworthy information but also a clear pathway for communication, facilitating active engagement and fostering a deeper understanding of ADRD-related information.

### Theme 3: The Crucial Role of the Community in ADRD-Related Information Dissemination and Evaluation

This theme highlights the pivotal role of the community in the dissemination and evaluation of information (Textbox 3). One significant finding was that participants frequently relied on their church as their most reputable source for ADRD information:

Churches are really getting more people aware of ADRD, and they are trying to help support people. I think the churches are getting more and more involved.

P3; chatbot

**The key findings concerning the multiple roles that the community plays in both information dissemination and evaluation.**

**Alzheimer disease and related dementias (ADRD) information dissemination**

- Churches and other physical communities are an informational hub
- Word of mouth is essential in ADRD information dissemination
- Sharing ADRD personal stories to look for information and support

**ADRD information evaluation**

- Community members help each other evaluate ADRD health professionals
- Community members help each other verify ADRD information
- Expertise is achieved with the help of the community

This observation underscores the significance of community-based physical places such as churches as central hubs in which ADRD-related discussions take place among members of the African American and Black community:

I think that in the Black community, a lot of information needs to come through the church because they trust the church.

P13; chatbot

Apart from the church, the dissemination of ADRD-related information occurs within the community through word of mouth. The act of sharing personal experiences of living with or caring for individuals with ADRD fosters a sense of solidarity among community members. By collectively seeking solutions and support, these individuals create a supportive network that encourages open discussions and knowledge exchange:

Word of mouth is also important. I talk to other people, asking their experience with ADRD, to find out about their experience, but also asking them if they can recommend a doctor, things like these.

P12; chatbot

Information is not only shared among members of a community but also assessed and evaluated collectively. Through word of mouth, the credentials of ADRD doctors and specialists are discussed within the community, and their expertise and information are shared among its members. Community members take on an active role in verifying the credibility and reliability of the information by engaging in conversations and comparing different sources:

First, I would look up more information about the doctors, see a little bit more about them: how long have they been practicing, different specialty areas, and then I would share that information with some of my family members, their caregivers and other members of my community, to see whether they know those specialists and or if the information is similar to what they already have, also to let them know specialists closer to them. I have family members in like Indianapolis, in Maine, Alabama, that have ADRD, so I just want to let them know when I found some other resources that they can connect to, if they need to.

P15; voice assistant

In certain instances, community-centered information holds significant value for participants as it aligns with their specific needs and interests. Participants expressed a strong desire for health technology that not only provides general ADRD health information but also offers insights into ADRD-related events and resources directly related to their physical community:

These are all opportunities to get more information: the community, what’s out in the community that could help you, because sometimes you might not have family support. It could be a single child dealing with a parent that has ADRD And that’s difficult. So you would need to find out what’s in the community for you.

P3; mobile app

This is not limited to community resources; rather, it includes knowing how living in a specific community might influence the development of ADRD:

I would like for the information to come from a source in this geographical area, just in case, there’s something that’s in this area, and that’s influencing how people develop ADRD, whether it could be the steel mills, or you know, pollution, or whatever it might be. So I would hope that information is provided and available is coming from this the area.

P14; mobile app

This theme highlights the crucial role of the community in health-related information seeking, not only in providing information but also in shaping the kind of information that individuals seek, particularly concerning ADRD.

### Theme 4: The Preference for a Direct, Time-Efficient, and Personified Interaction With Technology

The final theme delves into the participants’ strong preference for both natural conversation and direct interactions with technology (Textbox 4). For both the voice assistant and chatbot, participants expressed a preference for interactions that could resemble natural conversations:

Yeah, I think if Lola [voice assistant] introduces herself, and then you start talking and ask her the question and then get what general information she has, then you can write down what she says. Then, I think she should ask: “Is there anything else I can help you with?” If not, she says goodbye, or something like that.

P9; voice assistant

**The key findings concerning the characteristics of a perfect interaction.**

**The preference for a direct, time-efficient, and personified interaction with the technology**

The interaction must meet the following characteristics

- Be as natural as possible
- Avoid being intrusive
- Be direct and time efficient
- Have a tone of voice that resembles African American and Black voices
- Adapt to the user’s personality and needs

Participants described their interactions with the chatbot and voice assistant as reminiscent of popular systems such as Alexa and Siri, especially when referring to the voice assistant.

However, they pointed out a notable distinction in their perception of Lola the *voice assistant* and Lola the *chatbot*. They found both versions of Lola to be less intrusive compared to other mainstream systems such as Alexa and Siri. The participants’ preferences became evident as they emphasized the importance of avoiding excessive intrusiveness, valuing the ability of the system to disengage once the required information is provided:

She [Lola chatbot] wasn’t as pushy as I find Siri and other guys are. I was happy with that thing. Siri, and the other guys, they turn around: sometimes they will ask, you ask a question, they will continue to ask questions, even though you obtained the information wanted, but they keep asking questions.

P2; chatbot

One participant highlighted that the tone and sound of the voice were equally significant factors in shaping her perception of their interactions:

To me Lola [the voice assistant] sounds like somebody I could talk to. She kind of sounds like me. So that’s favorable for me. I do not like, I do not like calling these help desks for various companies, and getting people in foreign countries who I cannot understand. And they cannot understand me. So she was very clear. She spoke good English. And she spoke in a tone that I could hear, as well as understand. And that’s going to be great for people that are looking for information, especially older people.

P1; voice assistant

Moreover, participants deliberated on how much Lola should know about its users and their individual preferences. They expressed a strong desire for a customized system that could cater to their specific needs, adapting to their unique personalities, lifestyles, and even physical constraints. In this context, they envisioned a personalized tool capable of gathering sensitive information about users, such as their insurance details, to provide tailored and relevant assistance:

But there’s more information that I would need to get from her [Lola, voice assistant], and she couldn’t provide that information. I would like to ask her to give me more information on the doctors and to ask her if they accept my type of insurance, these kinds of things.

P9; voice assistant

In some cases, the level of customization included adapting to the user’s personality:

Hopefully, if I were using Lola [voice assistant]. And Lola was someone that was with me all the time, or around me most of the time. She would also pick up on my personality and my name. Like hello, P2. What can I do for you today?

P2; voice assistant

This participant’s quote reinforces the significance of a humanlike interaction in which both the user and the voice assistant learn from past interactions to establish a sense of familiarity and understanding. Interestingly, the participants did not express concerns about the voice assistant collecting too much data during these interactions.

On the other hand, the main insight concerning the mobile app that was gathered from the users was the creation of an interaction that would be more direct and to the point. An experience that was less designed around navigating through categories and subcategories and more built around a direct, search-based interaction was desired:

If there were clear headings, then I was going to find what I was looking for quicker, but since I was not really sure, I did not get right away what I wanted. I guess it could be quicker for me just to search it directly.

P6; mobile app

This final theme provided evidence of the pivotal role of designing interactions that are direct but also customized to the users’ needs, preferences, and constraints.

## Discussion

### Principal Findings

This research aimed to test the use of a voice assistant, chatbot, and mobile app with older African American and Black adults for seeking ADRD information.

First, participants emphasized the need to know the sources of health information and obtain contact details for organizations, doctors and specialists, likely due to historical mistrust of medical institutions [9-11]. In addition, significant accessibility barriers were identified, including small text fonts and overly complex user flows in the chatbot and mobile app. Low digital literacy and limited access to technology were also obstacles to adopting these tools.

Concerning the type of health information, participants sought community-relevant information. Moreover, we found that communities not only facilitated access to information but also collectively evaluated it. Major health decisions such as choosing an ADRD specialist were discussed within the community for collective solutions.

In interactions with the chatbot and voice assistant, participants valued a natural conversational flow with control over the interaction, such as slowing down or repeating information. In addition, participants perceived the study’s conversational agents as less intrusive and safer than commercial devices such as Siri or Alexa. Participants had fewer privacy concerns with the study’s custom-designed systems, possibly due to their awareness of the differences from commercial devices.

### Comparison With Prior Work

Our findings are partially consistent with those of previous studies even though our findings often expand beyond the findings observed. Concerning the need for the source of the information when presented with health information, our findings are consistent with those of previous studies with the same population [34,42].

Concerning the importance of accessible content and removing accessibility barriers, our study found similar barriers to those observed in previous research with older adults [54]. In addition, our study supports the findings of recent previous work [34], which highlighted the concern specific to older African American and Black adults that the reliance on technology might exclude some members of their community from accessing health interventions.

In line with previous research conducted on older African American adults [20,55], we found that participants seek community-relevant information. We expanded on these findings by showing that, for ADRD information, communities facilitated access to and collectively evaluated the information. The crucial role that the community plays in seeking ADRD health information appears to be a unique requirement within the African American and Black community. This stands out, especially when considering that previous research on other ethnic groups neither explicitly addresses nor mentions such a community-centric need [56,57]. These findings could be attributed to the unique collectivist nature of African American and Black communities, which has already been highlighted in some studies [20]. However, previous research has not explored in depth how the role of the community relates to ADRD health information seeking.

Our research builds on studies on voice assistants [56,58] that show users’ desire for agency and control over conversational agents. Our chatbot and voice assistant were seen as less intrusive and safer compared to Siri, Google, or Alexa. This differs from previous studies using commercial devices, raising questions about whether these findings are unique to our sample or more broadly applicable [49,56,58].

The use of the Wizard of Oz methodology ensured that our voice assistant always understood and addressed participants’ requests, which may have influenced their positive evaluations. Previous studies have shown that commercial voice assistants often fail to understand older African American adults, leading to frustration and the need for code switching, a mentally demanding requirement not necessary with our system [59-61].

### Practical Design Implications

#### Overview

Our study has implications for how to design ADRD technology tailored for African American and Black communities. In particular, our findings provide novel inputs on both the specific type of ADRD information that should be incorporated into a technology tailored for this specific population and how the interaction should be constructed to be tailored and relevant for this population.

#### Designing Credible and Accessible Content

Given the history of racial health discrimination [35,36] and ongoing inequities in health care for African American and Black people [37], it is crucial for ADRD technologies to provide clear and accurate information about sources. This can be achieved by including details such as the names of the agencies, laboratories, associations, and organizations that provided the information (eg, Alzheimer’s Association or National Institutes of Health). Given that associations such as the Alzheimer’s Association have local chapters, contact information of a representative of the associations could be provided to the user if requested.

Considering the role of the community in ADRD information seeking and evaluation, it is essential to provide community-relevant health information. This includes details on local ADRD events, support groups, and financial resources available in the users’ area.

In addition, information on how lifestyle factors specific to the users’ geographical area can impact ADRD development and treatment should be included. By integrating these elements, ADRD technologies can better meet the needs of African American and Black communities, providing them with reliable, accessible, and relevant health information.

In addition, given their age (>60 y), some older adults might have vision or hearing limitations. Hence, making sure that the font is big enough for them to read the content and that the voice assistant’s volume can be regulated to meet the users’ needs is important. In addition, given the low health literacy, which is common among this population [12,24], it proves to be essential when designing ADRD technological interventions to make sure that the language is clear and concise and avoids medical jargon.

#### Designing a Technological Intervention That Fosters Users’ Representation and Control

Some participants mentioned that elements such as the voice of the voice assistant could impact the ADRD tool’s perceived trustworthiness. Some studies have attempted to add a race-related component to conversational agents [42] and found discording results. Future designs could attempt to uncover whether creating an ADRD technology that people can feel comfortable with and can perceive as familiar to them (by providing a familiar voice, including pictures and images coming from their community, or including messages from people they know) might increase the technology’s perceived trustworthiness and its related adoption and use.

Moreover, our study showed that a system capable of clearly understanding participants might lead to more perceived trustworthiness and more potential future use and adoption. Given the importance of eliciting trust in this population to then foster adoption and use, it is important when developing future voice assistants meant for ADRD to acknowledge multiple ways of speaking a language and that participants not be asked to change their natural ways of speaking to access a health technology intervention.

In addition, when designing voice assistants providing ADRD-related information, making sure that the user feels in control of the interaction and not overwhelmed by it, giving the user the chance to ask the technology to slow down and repeat itself if necessary, is significant for increasing accessibility. This could be true especially for caregivers of people diagnosed with ADRD, who often find themselves in overwhelming environments with cognitive and time constraints. In addition, concerning mobile apps and chatbots, given that ADRD represents a health concern for most people aged >65 years, it is important that the font size is large enough for users to see and does not present a barrier to use.

#### Designing a Technological Intervention That Is Time Efficient

Our findings show a preference for efficient interactions with all modalities. Participants wanted to obtain information quickly without navigating through multiple categories (mobile app) or answering numerous questions before obtaining relevant information (voice assistant and chatbot). This likely reflects the time constraints faced by ADRD caregivers who need to access useful information promptly.

Efficiency could be improved by creating more humanlike conversational agents and designing technologies that personalize user experiences. For instance, tools could learn users’ demographic information and health insurance details to help them locate ADRD specialists within their geographical area and insurance network.

Personalization through data collection was not a privacy concern for our participants. However, future designs should address potential privacy and security concerns by ensuring effective data protection and informing users about data collection practices. Given the historical marginalization of this population, it is crucial that privacy remains a guiding principle in any future implementations.

#### Designing a Technological Intervention That Leverages Preexisting Community Processes

When designing an ADRD-specific technology, we recommend that future designs take into consideration the need for older African American and Black adults to save the information obtained from the tool to share it with their community and create their own physical and digital repository of ADRD-related information. Therefore, we suggest that future designs construct ADRD technology that offers users the opportunity to print and share pieces of information and tools to facilitate the creation of their personalized ADRD information repositories.

In addition, given the collectivist information evaluation process unique to African American and Black older adults, it proves to be important to offer them opportunities to discuss health-related information with their peers, such as doctors or practitioners working in the field of ADRD or associations providing help for ADRD caregivers. Given that it would be impossible to recreate a physical, collective space as influential as the church for this specific population, it is significant that future designs create tools built on preexisting community information structures and support community dynamics and practices.

Finally, participants pointed out how low digital literacy within their community could be a barrier to adopting an ADRD-related technological intervention, with older African American and Black adults worrying that some members of their community might not have the economic resources and digital expertise to access the tool and obtain the benefits from it.

While this could be seen as an insurmountable obstacle for adopting technological interventions, it represents new opportunities for the HCI community. In fact, designing an ADRD-related technology for older African American and Black adults might become a way to implement technological health interventions paired with physical solutions or design an infrastructure of technological and human-mediated support for people with limited access to digital tools.

### Limitations and Future Work

This study has 3 major limitations worth noting. First, the number of participants was limited, with most (10/15, 67%) identifying as women. This could have impacted the findings observed. To ensure broader applicability, future research should strive for a larger and more diverse sample including a balanced representation of gender identities and other demographic factors.

In addition, testing was conducted in a controlled setting (a room with only the participant and researchers present), which might have influenced the findings. Moreover, this study might have been influenced by the observer effect given the research design. To address this limitation, future studies could explore testing in more naturalistic settings that better resemble real-world use scenarios where participants may feel less observed or pressured.

Finally, one last observation has to be made regarding testing. Considering that it was a Wizard of Oz study manipulated by a researcher, the voice assistant did not show any issue in understanding our participants’ speech. Thus, the fact that the voice assistant was always able to clearly understand and address participants’ requests might have impacted the way in which they evaluated and related to the tool. In our case, given the specific methodology used, engaging with the voice assistant did not require the participants to engage in code switching but allowed a vast variety of dialects and versions of English to be understood. This lack of need for code switching or linguistic adaptation may have positively influenced participant trust in and engagement with the technology, potentially skewing their perceptions compared to interactions with commercially available voice assistants.

### Conclusions

This research tested 3 different technological modalities using a Wizard of Oz methodology: a mobile app, a voice assistant, and a chatbot designed for older African American and Black adults. The goal of this health technology intervention was to provide participants with accurate and reliable ADRD information. Our findings show that several factors are involved in creating meaningful and successful technologies, such as the importance of providing sources for the information, adopting a community-centered approach, and creating an interaction that appears to be as natural as possible.

This study offers new insights on designing health technology for this underserved population by highlighting the importance of tailoring the design of a technology to satisfy the culturally specific needs of a population and using a user-centered approach to create inclusive and accessible solutions.

Overall, this study contributes to the growing body of research in the field of health technology and HCI, emphasizing the significance of user-centered design and cultural sensitivity in developing innovative solutions to bridge health disparities and empower older African American and Black adults in their health care journeys.

## Abbreviations

ADRD: Alzheimer disease and related dementias
HCI: human computer interaction

## Data Availability

The datasets generated during and analyzed during this study are available upon request.
